# Effects of premedication with atropine, penehyclidine, and glycopyrrolate on postoperative delirium in older patients undergoing endoscopic gastric or esophageal submucosal dissection: a randomized, double-blind, multicenter, controlled clinical trial

**DOI:** 10.3389/fphar.2026.1905662

**Published:** 2026-08-21

**Authors:** Wen Lin Xian, Jing Peng, Qinxian Wei, Na Wang, Xiao Pei Gao, Sisi Zeng, Qin Ye, Wu Chang Fu, Fang Jun Wang

**Affiliations:** 1 The Affiliated Hospital, North Sichuan Medical College, Nanchong, China; 2 The First People’s Hospital of Liangshan Yi Autonomous Prefecture, Xichang, China; 3 The Third People’s Hospital of Chengdu, Chengdu, China; 4 The Second Affiliated Hospital of the Chinese Army Medical University, Chongqing, China; 5 The Zigong Fourth People’s Hospital, Zigong, China; 6 The Second Clinical Medical Institution of North Sichuan Medical College (Nanchong Central Hospital), Nanchong, China

**Keywords:** atropine, glycopyrrolate, older, penehyclidine, postoperative delirium, premedication

## Abstract

**Background:**

Anticholinergic drugs are commonly used as premedication in clinical practice. The effects of premedication of atropine, penehyclidine, and glycopyrrolate on postoperative delirium in older patients remain unclear.

**Objective:**

The aim of this study was to observe the effects of premedication with atropine, penehyclidine, and glycopyrrolate on postoperative delirium (POD) in older patients undergoing endoscopic gastric or esophageal submucosal dissection.

**Methods:**

Older patients undergoing endoscopic gastric or esophageal submucosal dissection were randomly divided into atropine group (A group), glycopyrrolate group (G group), penehyclidine group (P group), and normal saline group (NS group), with 144 cases in each group. The primary outcome was the incidence of POD 24 h after surgery. The secondary outcomes were the incidence of POD 48 h and 72 h after surgery, perioperative saliva secretion levels, RASS scores, QoR-15 scores, VAS scores of dry mouth, postoperative nausea VAS scores, and severity of postoperative vomiting.

**Results:**

The incidence of POD 24 h after surgery was significantly higher in the A (20.1%) and P (23.6%) groups than in the NS group (9%) (95% CI, 0.030–0.191, *p* = 0.006, and 95% CI, 0.062–0.229, *p* = 0.001, respectively), and the incidence of POD between the NS group (9.0%) and G group (10.4%) was comparable (95% CI, −0.054–0.082, *p* = 0.843). Oral saliva secretion in the A, G, and P groups at T_1_–T_5_ was significantly lower than that in the NS group (all *p* < 0.001). Twenty four hours after surgery, the QoR-15 score in the A group (124.0 ± 13.3) was significantly lower than that in the NS group (132.4 ± 12.1) (95% CI, −11.338 to −5.397, *p* < 0.001).

**Conclusion:**

Glycopyrrolate is more suitable as a premedication for older patients without aggravating postoperative delirium or prolonging dry mouth.

**Clinical Trial Registration:**

http://www.chictr.org.cn/showproj.html?proj=212300; Registration number: ChiCTR2300078413.

## Background

Anticholinergic drugs are commonly used as premedication in clinical practice, acting on central and peripheral nerve postganglionic cholinergic receptors, reducing glandular secretion, relieving various smooth muscle tensions, maintaining airway patency, preventing postoperative pulmonary complications, preventing bradycardia caused by laryngeal stimulation during intubation and vagus nerve reflex, and antagonizing bradycardia caused by various anesthetics.

Atropine can non-selectively block multiple subtypes of M receptors, glycopyrrolate mainly selectively blocks M_3_ receptors, and penehyclidine selectively blocks M_1_ and M_3_ receptors. Clinically, all of them are used as premedication, which can effectively inhibit saliva secretion and facilitate surgical procedures (Kim et al., 2017; Yao et al., 2020; Liu et al., 2019). Anticholinergic drugs can penetrate the blood–brain barrier and act on central cholinergic receptors, affecting the central nervous system and resulting in cognitive side effects. The administration of the combination of neostigmine/atropine at the end of surgery for reversing neuromuscular blockade leads to postoperative cognitive dysfunction (POCD) in patients undergoing elective surgery (Batistaki et al., 2017). Intramuscular injections of penehyclidine 5 min before induction of anesthesia increased the incidence of postoperative cognitive dysfunction and postoperative delirium (POD) in older patients undergoing thoracoscopic radical surgery for lung cancer under general anesthesia (Hongyu et al., 2019). Due to the inability of glycopyrrolate to penetrate the blood–brain barrier, its central nervous system side effects are relatively few (Li et al., 2022). We speculated that glycopyrrolate is more suitable for preoperative medication in older patients than atropine and penehyclidine without exacerbating POCD. At present, there are no studies on the effects of premedication with atropine, penehyclidine, and glycopyrrolate on postoperative delirium in older patients. Therefore, we conducted this clinical trial to observe the effects of premedication with atropine, penehyclidine, and glycopyrrolate on postoperative delirium in older patients undergoing endoscopic gastric or esophageal submucosal dissection.

## Methods

The present controlled clinical study was approved by the Medical Ethics Committee of the Affiliated Hospital of North Sichuan Medical College (2022ER341-1) and by the medical ethics review committee at each participating center and registered in the Chinese Clinical Trial Registry (http://www.chictr.org.cn/showproj.html?proj=212300; registration number: ChiCTR2300078413, date of registration: 07/12/2023). The independent data monitoring and ethics committee and trial steering committee provided trial supervision, and the safety data and accruing outcomes were reviewed twice throughout the trial. All participants signed written informed consent prior to enrollment. Older patients scheduled for elective endoscopic gastric or esophageal submucosal dissection between December 2023 and July 2024 were included in this study. The inclusion criteria were as follows: an American Society of Anesthesiologists physical status of 1, 2, or 3; an age of 60–75 years; a body mass index (BMI) of 18.5–28 kg/m^2^ (Chen et al., 2023); and a diagnosis of early-stage esophageal or gastric cancers (Al-Haddad et al., 2023). The exclusion criteria included bronchial asthma; salivary gland dysfunction or dry mouth syndrome; glaucoma; prostate enlargement; pyloric obstruction; a history of allergy to anticholinergic drugs; liver, kidney, or cardiac dysfunction; recent participation in clinical trials of other drugs; a history of mental disorders and family history of mental illness; hearing impairment or inability to communicate normally; preoperative cognitive dysfunction or delirium; moderate-to-severe traumatic brain injury within 3 months before surgery; and refusal by patients or relatives to sign written informed consent. The withdrawal criteria were the duration of surgery >4 h, a change in the surgical plan, allergy to drugs, and incomplete data collection.

A total of 576 patients who participated in this clinical trial were randomly assigned to the atropine group (A group), glycopyrrolate group (G group), penehyclidine group (P group), and normal saline group (NS group) in a ratio of 1:1:1:1. Randomization was conducted by an anesthesia assistant independently in each center, who was not responsible for anesthesia of the older patients, data collection, and statistical analysis in this trial, using the random number table. The grouping information of all patients was marked on the cards and sealed in the opaque envelopes. Thirty minutes before surgery, an anesthesiologist sequentially selected the sealed opaque envelope and prepared the premedication (atropine 0.01 mg/kg, glycopyrrolate 0.005 mg/kg, penehyclidine 0.01 mg/kg, and normal saline) according to the group assignment indicated on the card sealed in the opaque envelope; all drugs were diluted to 2 mL with normal saline and then administered intramuscularly by surgical ward nurses who were blinded to the grouping information. The digestive physician, participating patients, operating room and ward nurses, anesthetists responsible for anesthesia, indicator evaluators, and statistical analysts were blinded to the group assignment. The primary outcome was the incidence of POD 24 h after surgery. The secondary outcomes were the incidence of POD 48 h and 72 h after surgery, perioperative saliva secretion levels, RASS scores, QoR-15 scores, VAS scores for dry mouth and postoperative nausea, and severity of postoperative vomiting. All the anesthesia nurses who evaluated the POD in each center were trained in the psychiatry department of the affiliated hospital of North Sichuan Medical College by a psychiatrist with 10 years of clinical practice experience.

All patients were fasted preoperatively, with no solid food for 8 h and no fluids for 4 h, and received intramuscular premedication 30 min before surgery. Upon arrival at the operating room, patients were provided 5 L/min oxygen via a face mask and underwent routine monitoring of heart rate, electrocardiogram, respiratory rate, non-invasive blood pressure, end-tidal carbon dioxide, pulse oxygen saturation (SpO_2_), body temperature, and bispectral index. After establishing reliable peripheral intravenous (IV) access, compound sodium chloride solution was administered intravenously at a rate of 8–10 mL/kg·h during surgery. General anesthesia was induced with 0.04 mg/kg midazolam, 0.4 µg/kg sufentanil, 2 mg/kg propofol, and 0.8 mg/kg rocuronium intravenously. All patients underwent mechanically controlled ventilation after tracheal intubation. The tidal volume was 8–10 mL, the respiratory rate was 10–14 times/min, and the end-tidal carbon dioxide partial pressure was maintained at 35–45 mmHg. Sevoflurane (2%–3%) was continuously inhaled during surgery to maintain a bispectral index (BIS) value of 45–60. Intraoperative intravenous infusion of 10 mg of rocuronium and 10 µg of sufentanil was administered if required. All submucosal dissection procedures were performed by a digestive physician with more than 5 years of clinical experience. The administration of rocuronium and sevoflurane was stopped 45 and 5 min before the end of surgery, respectively. After extubation, the patient was transferred to the post-anesthesia care unit (PACU) for continuous nasal catheter oxygen inhalation and vital sign monitoring. If the Steward score was ≥4, the patient was sent back to the ward.

The Confusion Assessment Method (CAM) scale was applied the day before surgery and 24, 48, and 72 h after surgery by an anesthesia nurse who was blinded to the group assignment. The CAM scale includes the following contents: (1) acute onset and fluctuating course; (2) inattention; (3) disorganized thinking; and (4) altered levels of consciousness. If both (1) and (2), along with either (3) or (4), are presented, POD can be determined to have occurred (Aoki et al., 2023). The Saxon test was used to evaluate the patient’s oral saliva secretion before administration of premedication (T_0_), 5 min before anesthesia (T_1_), 5 min before surgery (T_2_), at the end of surgery (T_3_), and 2 h (T_4_), 8 h (T_5_), and 24 h (T_6_) after surgery. The patient chewed two pieces of sterile gauze for 2 min, and then saliva secretion was measured based on the change in gauze weight before and after chewing (Minagi et al., 2022). The Richmond Agitation Sedation Scale (RASS: +4, combative; +3, very agitated; +2, agitated; +1, restless; 0, alert and calm; −1, drowsy; −2, light sedation; −3, moderate sedation; −4, deep sedation; −5, unarousable) (Wang et al., 2022) was used by an anesthesia nurse who was unaware of the group assignment to evaluate the PACU emergence agitation of patients 10 min (t_1_), 20 min (t_2_), 30 min (t_3_), and 60 min (t_4_) after extubation. The total QoR-15 scores of patients were evaluated 24, 48, and 72 h postoperatively. The occurrence of dry mouth and postoperative nausea in the four groups was evaluated 1 h before surgery and 2, 8, 24, and 48 h after surgery using the VAS score. The severity of postoperative vomiting in patients was evaluated according to a numeric rank score (0, no nausea or vomiting; 1, nausea but no vomiting; 2, vomiting once or twice; 3, vomiting on more than two occasions) (Oh et al., 2010).

### Statistical analysis

We used PASS15.0 to calculate the sample size based on a preliminary experiment with 20 patients in each group. The incidences of POD in A, G, P, and NS groups were 20%, 2%, 25%, and 10%, respectively, 24 h after surgery. A sample size of 115 patients would be required in each group with a type I error of 0.05 and power of 0.9. Considering a 20% dropout rate, there should be 144 cases in each group.

SPSS 26.0 statistical software was used for statistical analysis. Continuous measures with a normal distribution were presented as mean ± standard deviation (SD) and analyzed using Student’s t-test, whereas continuous measures with a non-normal distribution were presented as median [interquartile range (IQR)] and analyzed using the Kruskal–Wallis test. The chi-square test or Fisher’s exact test was used to analyze counting and categorical data, which were presented as numbers (%). Continuous variables at different time points were analyzed using a two-way repeated measurement ANOVA, and count and categorical data at different time points were analyzed using generalized estimating equations. If the interaction between groups and time was positive, the differences in outcomes between groups at different time points were analyzed using Bonferroni’s correction. A *p*-value less than 0.05 was considered statistically significant.

## Results

**FIGURE 1:**
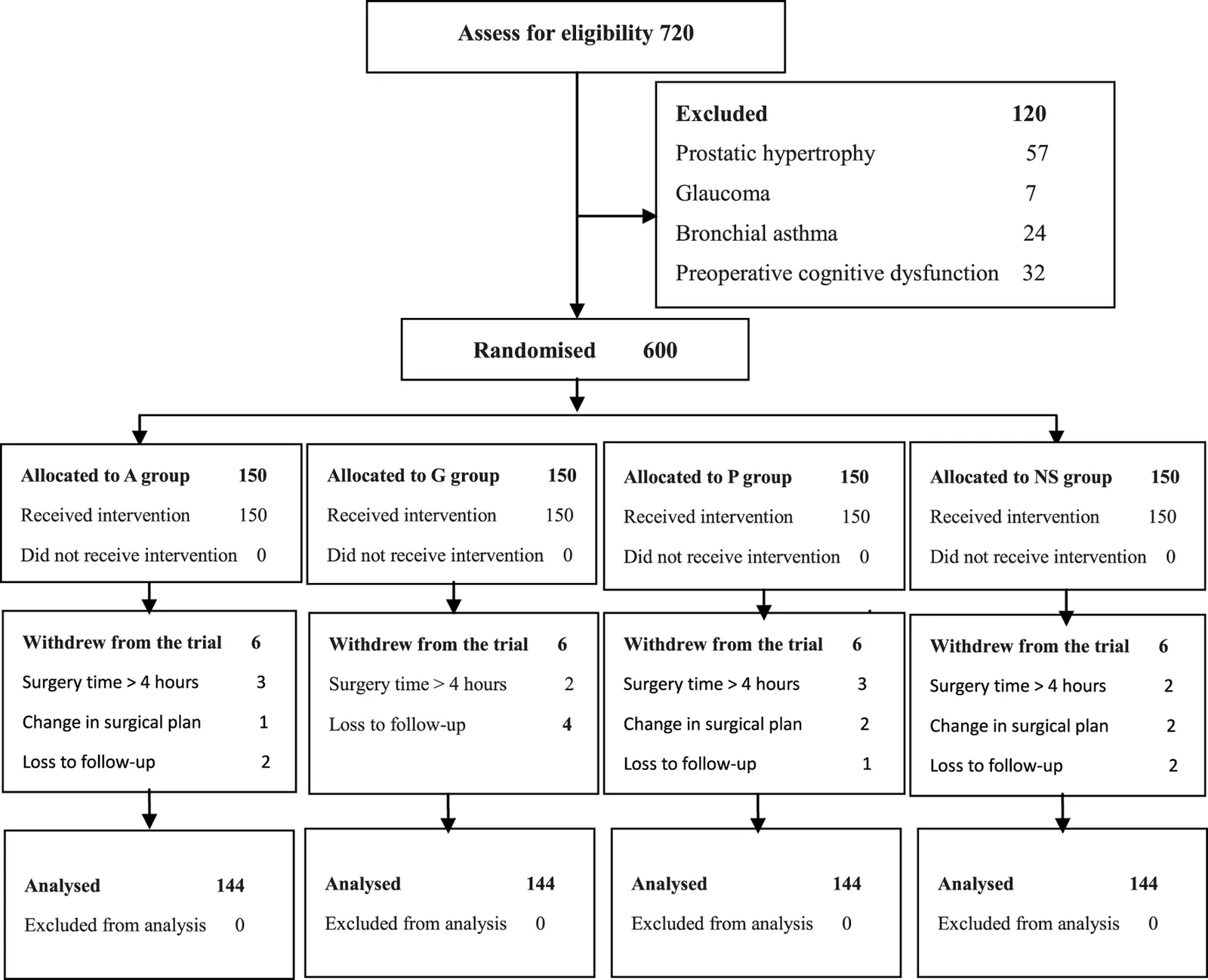
Participant flow diagram.

A total of 720 older patients were screened for eligibility. Fifty-seven patients with prostatic hypertrophy, 7 patients who had glaucoma, 24 patients with bronchial asthma, and 32 patients with preoperative cognitive dysfunction were excluded. A total of 600 patients were subsequently allocated to four groups. Ten patients with surgery time >4 h, nine patients with a change in surgical plan, and five patients who were lost to follow-up were withdrawn from the trial. A total of 576 patients completed the study (Figure 1).

The demographic data in four groups are shown in Table 1. There were no significant differences in age, weight, height, ASA class, education level, gender ratio, types of endoscopic mucosal dissection, dosage of sufentanil, surgery time, duration of anesthesia, and hospitalization time among the four groups (*p* = 0.225, 0.683, 0.321, 0.395, 0.883, 0.674, 0.367, 0.218, 0.130, and 0.310, respectively). The duration of PACU was longer in the A and P groups than in the NS group (95% CI, 2.433–5.191, *p* < 0.001, and 95% CI, 1.389–3.958, *p* < 0.001, respectively), whereas the duration of PACU was shorter in the G and NS groups than in the A group (95% CI, −4.860–1.820, *p* < 0.001, and 95% CI, −5.191–2.433, *p* < 0.001, respectively).

**TABLE 1:** Demographic data in four groups.

Index	A group (n = 144)	G group (n = 144)	P group (n = 144)	NS group (n = 144)	F/χ^2^ values	*p*-values
Age, mean ± SD (y)	67.9 ± 4.0	68.1 ± 3.7	67.5 ± 3.9	67.2 ± 4.2	1.457	0.225
Weight, mean ± SD (kg)	62.4 ± 6.5	63.0 ± 6.8	62.7 ± 7.0	63.4 ± 6.6	0.499	0.683
Height, mean ± SD (cm)	163.2 ± 6.8	162.1 ± 7.0	162.9 ± 6.6	161.8 ± 7.2	1.170	0.321
Gender, *n* (%)						
Female	52 (36.1)	43 (29.9)	45 (31.3)	49 (34)	1.536	0.674
Male	92 (63.9)	101 (70.1)	99 (68.7)	95 (66)		
ASA class, *n* (%)						
I	26 (18.1)	28 (19.4)	30 (20.8)	29 (20.1)	1.084	0.982
II	85 (59.0)	87 (60.5)	80 (55.6)	84 (58.4)		
III	33 (22.9)	29 (20.1)	34 (23.6)	31 (21.5)		
Educational level, *n* (%)						
Primary school	36 (25.0)	29 (20.1)	32 (22.2)	39 (27.1)	2.313	0.889
Middle school	69 (47.9)	72 (50.0)	70 (48.6)	67 (46.5)		
More than middle school	39 (27.1)	43 (29.9)	42 (29.2)	38 (26.4)		
Types of endoscopic mucosal dissection, *n* (%)						
Endoscopic esophageal mucosal dissection	98 (68.1)	89 (61.8)	93 (64.6)	84 (58.3)	3.165	0.367
Endoscopic gastric mucosal dissection	46 (31.9)	55 (38.2)	51 (35.4)	60 (41.7)		
Dosage of sufentanil, mean ± SD (μg)	24.7 ± 3.3	24.9 ± 63.1	25.2 ± 3.6	25.5 ± 3.4	1.482	0.218
Surgery time, mean ± SD (min)	52.8 ± 6.4	54.0 ± 6.6	53.0 ± 5.9	54.1 ± 5.2	1.893	0.130
Duration of anesthesia, mean ± SD (min)	63.8 ± 6.8	63.9 ± 6.5	63.1 ± 6.1	64.5 ± 4.7	1.196	0.310
Duration of PACU, mean ± SD (min)	48.1 ± 6.7*	44.8 ± 6.4^#^	47.0 ± 5.9* ^,^ ^#^	44.3 ± 5.1^#^	12.784	<0.001
Hospitalization time, mean ± SD (d)	7.5 ± 1.1	7.3 ± 1.4	7.6 ± 1.5	7.7 ± 1.3	2.247	0.082

Values are presented as the mean ± SD and the number of patients. A group, atropine group; G group, glycopyrrolate group; P group, penehyclidine group; NS group, normal saline group; ASA, American Society of Anesthesiologists; PACU, post-anesthesia care unit. Differences in weight, age, height, and surgery time among four groups were analyzed using one-way ANOVA, and differences in gender, ASA class, educational level, and types of endoscopic mucosal dissection among four groups were analyzed using *Chi-*square tests. The Bonferroni correction test was used for further statistical comparisons between groups.

* *p* < 0.008 vs. NS group.

^#^ *p* < 0.008 vs. A group.

The incidence of POD in the four groups at 24 h, 48 h, and 72 h after surgery was significantly increased (*p* = 0.031, 0.037, 0.010, and 0.003, respectively). Twenty-four hours after surgery, the incidence of POD was increased more significantly in the A (20.1%) and P (23.6%) groups than in the NS group (9.0%) (95% CI, 0.030–0.191, *p* = 0.006, and 95% CI, 0.062–0.229, *p* = 0.001, respectively), and the incidence of POD between the NS group (9.0%) and the G group (10.4%) was comparable (95% CI, −0.054–0.082, *p* = 0.843). There was no difference in the incidence of POD among the four groups at 48 h and 72 h after surgery (*p* = 0.163 and 0.586, respectively), as shown in Table 2.

**TABLE 2:** Incidence of POD in the four groups.

Time points	A group (*n* = 144)	G group (*n* = 144)	P group (*n* = 144)	NS group (*n* = 144)	*χ* ^2^ values	*p*-values
The day before surgery, *n* (%)	0 (0)	0 (0)	0 (0)	0 (0)	-	-
24 h after surgery, *n* (%)	29 (20.1)* ^,^ ^#^	15 (10.4)^#^	34 (23.6)* ^,^ ^#^	13 (9.0)^#^	16.744	0.001
48 h after surgery, *n* (%)	12 (8.3)^#^	6 (4.2)^#^	15 (10.4)^#^	8 (5.6)^#^	5.121	0.163
72 h after surgery, *n* (%)	8 (5.6)^#^	5 (3.5)^#^	10 (6.9)^#^	6 (4.2)^#^	2.142	0.586
Wald χ^2^ values	4.653	4.345	6.692	9.104	-	-
P-values	0.031	0.037	0.010	0.003	-	-

Values are presented as the number of patients. A group, atropine group; G group, glycopyrrolate group; P group, penehyclidine group; NS group, normal saline group. Differences in the incidence of POD among four groups at different time points were analyzed using *Chi-*square tests or Fisher’s exact tests, while the incidence of POD at different time points within-group was analyzed using generalized estimating equations, and the Bonferroni correction test was used for further statistical comparisons between groups.

* *p* < 0.008 vs. NS group

^#^ *p* < 0.008 vs. the day before surgery.

Oral saliva secretion in the four groups at different time points is shown in Table 3. As the data for oral saliva secretion in the four groups violated sphericity (*p* < 0.001), a two-way repeated-measures analysis of variance with Greenhouse–Geisser correction was used. Oral saliva secretion in the A, G, and P groups at T_1_–T_5_ and in the NS group at T_1_–T_4_ was significantly decreased compared with that at T_0_ (all *p* < 0.001). Oral saliva secretion in the A, G, and P groups at T_1_–T_5_ was significantly lower than that in the NS group (all *p* < 0.001). Oral saliva secretion at T_1_–T_5_ in the G group and T_1_ and T_3_–T_5_ in the P group was significantly lower than that in the A group (*p* < 0.008), as shown in Table 3.

**TABLE 3:** Oral saliva secretion in the four groups.

Time points	A group (n = 144)	G group (n = 144)	P Group (n = 144)	NS group (n = 144)	*F*-value	*p*-values
T_0_	6.2 ± 1.4	6.3 ± 1.5	6.1 ± 1.3	6.2 ± 1.5	0.472	0.702
T_1_	1.7 ± 0.9* ^,^ ^#^	1.2 ± 0.8* ^,^ ^#^ ^,^ ^$^	1.3 ± 0.7* ^,^ ^#^ ^,^ ^$^	5.3 ± 1.3^#^ ^,^ ^$^	616.372	<0.001
T_2_	1.5 ± 0.6* ^,^ ^#^	1.2 ± 0.6* ^,^ ^#^ ^,^ ^$^	1.3 ± 0.5* ^,^ ^#^	5.1 ± 1.4^#^ ^,^ ^$^	686.090	<0.001
T_3_	1.6 ± 0.3* ^,^ ^#^	1.2 ± 0.5* ^,^ ^#^ ^,^ ^$^	1.1 ± 0.4* ^,^ ^#^ ^,^ ^$^	5.2 ± 1.4^#^ ^,^ ^$^	976.190	<0.001
T_4_	1.6 ± 0.4* ^,^ ^#^	1.3 ± 0.5* ^,^ ^#^ ^,^ ^$^	1.2 ± 0.4* ^,^ ^#^ ^,^ ^$^	5.5 ± 1.3^#^ ^,^ ^$^	1,084.565	<0.001
T_5_	4.3 ± 1.1* ^,^ ^#^	3.3 ± 1.0* ^,^ ^#^ ^,^ ^$^	2.4 ± 1.2* ^,^ ^#^ ^,^ ^$^	6.5 ± 1.3^$^	316.099	<0.001
T_6_	6.2 ± 1.5	6.3 ± 1.4	6.4 ± 1.7	6.2 ± 1.4	0.686	0.561
*F*-values	714.575	844.062	800.255	22.918	-	-
*P* values	<0.001	<0.001	<0.001	<0.001	-	-

Values are presented as the mean ± SD. A group, atropine group; G group, glycopyrrolate group; P group, penehyclidine group; NS group, normal saline group. T_0_, before premedication; T_1_, 5 min before anesthesia; T_2_, 5 min before surgery; T_3_, the end of surgery; T_4_, 2 h after surgery; T_5_: 8 h after surgery; T_6_: 24 h after surgery. Differences in the oral saliva secretion among the four groups were analyzed using a two-way repeated measurement ANOVA with Greenhouse–Geisser correction, and the Bonferroni correction test was used for further statistical comparisons between groups.

* *p* < 0.008 vs. NS, group

^#^ *p* < 0.003 vs. T_0_

$ *p* < 0.008 vs. A group.

The RASS scores in the four groups at different time points are shown in Table 4. There was no difference in the RASS scores at t_1_–t_4_ among the four groups (*p* = 0.900, 0.996, 0.802, and 0.984, respectively).

**TABLE 4:** RASS scores in four groups at different time points after extubation.

Time point	Group	n	−1	0	1	2	*χ* ^2^ values	*p*-values
t_1_	A group	144	46	46	33	19	4.174	0.900
	G group	144	47	45	35	17		
	P group	144	54	40	28	22		
	NS group	144	53	37	36	18		
t_2_	A group	144	48	42	31	23	1.656	0.996
	G group	144	50	44	29	21		
	P group	144	52	44	28	20		
	NS group	144	54	37	30	23		
t_3_	A group	144	44	46	32	22	5.354	0.802
	G group	144	39	55	35	15		
	P group	144	39	54	34	17		
	NS group	144	49	47	28	20		
t_4_	A group	144	5	90	49	0	1.138	0.984
	G group	144	4	92	48	0		
	P group	144	6	86	52	0		
	NS group	144	6	91	47	0		

Values are presented as the number of patients. A group, atropine group; G group, glycopyrrolate group; P group, penehyclidine group; NS group, normal saline group; t_1_, 10 min after extubation; t_2_, 20 min after extubation; t_3_, 30 min after extubation; t_4_, 60 min after extubation. Differences in the RASS scores among the four groups were analyzed using *Chi-*square tests or Fisher’s exact tests.

The postoperative QoR-15 scale scores of patients in four groups are shown in Table 5. At 24 h after surgery, the QoR-15 score in the A group was significantly lower than that in the NS group (95% CI, −11.338 to −5.397, *p* < 0.001). At 48 h and 72 h after surgery, the QoR-15 scores in the A group were significantly higher than those at 24 h after surgery (95% CI, 3.858–10.641, *p* < 0.001, and 95% CI, 8.199–14.981, *p* < 0.001). At 72 h after surgery, the QoR-15 scores were significantly higher than those at 24 h after surgery in the G group (95% CI, 3.519–10.397, *p* < 0.001), P group (95% CI, 5.567–11.835, *P* < 0.001), and NS group (95% CI, 2.492–8.826, *p* < 0.001).

**TABLE 5:** Postoperative QoR-15 scale scores in the four groups.

Time points	A group (n = 144)	G group (n = 144)	P Group (n = 144)	NS group (n = 144)	*F* value	*p-*values
24 h after surgery	124.0 ± 13.3^*^	130.4 ± 14.1	128.0 ± 13.6	132.4 ± 12.1	10.455	<0.001
48 h after surgery	131.2 ± 11.7^#^	133.1 ± 13.1	132.0 ± 11.3	134.2 ± 12.2	1.637	0.180
72 h after surgery	135.6 ± 10.6^#^	137.3 ± 8.3^#^	136.7 ± 7.3^#^	138.0 ± 8.7^#^	1.982	0.116
*F*-values	34.443	11.995	22.308	9.605	-	-
*P*-values	<0.001	<0.001	<0.001	<0.001	-	-

Values are presented as the mean ± SD. A group, atropine group; G group, glycopyrrolate group; P group, penehyclidine group; NS group, normal saline group. Differences in the QoR-15 scale scores among four groups were analyzed using a two-way repeated measurement ANOVA with Greenhouse–Geisser correction, and the Bonferroni correction test was used for further statistical comparisons between groups.

* *p* < 0.008 vs. NS group

^#^ *p* < 0.017 vs. 24 h after surgery.

The VAS scores of dry mouth in patients at 1 h before surgery and at 2 h, 8 h, 24 h, and 48 h after surgery in the four groups are shown in Table 6. The VAS scores of dry mouth at 2, 8, and 24 h after surgery were significantly higher in the A, G, and P groups than in the NS group (all *p* < 0.001).

**TABLE 6:** VAS scores of dry mouth in the four groups.

Time point	A group (n = 144)	G group (n = 144)	P Group (n = 144)	NS group (n = 144)	*F*-values	*p*-values
1 h before surgery	0.7 ± 0.6	0.6 ± 0.7	0.5 ± 0.6	0.6 ± 0.6	1.103	0.347
2 h after surgery	6.0 ± 0.8* ^,^ ^#^	6.1 ± 0.9* ^,^ ^#^	6.2 ± 0.8* ^,^ ^#^	2.1 ± 0.9^#^	747.213	<0.001
8 h after surgery	4.1 ± 0.9* ^,^ ^#^	5.2 ± 1.1* ^,^ ^#^	6.3 ± 1.0* ^,^ ^#^	1.8 ± 1.1^#^	480.657	<0.001
24 h after surgery	2.5 ± 0.6* ^,^ ^#^	2.6 ± 0.7* ^,^ ^#^	4.5 ± 0.6* ^,^ ^#^	1.6 ± 0.7^#^	505.918	<0.001
48 h after surgery	0.8 ± 0.7	0.7 ± 0.6	0.6 ± 0.6	0.7 ± 0.6	2.247	0.082
*F*-values	1310.227	1367.143	2028.946	95.941	-	-
*P*-values	<0.001	<0.001	<0.001	<0.001	-	-

Values are presented as the mean ± SD. A group, atropine group; G group, glycopyrrolate group; P group, penehyclidine group; NS group, normal saline group. Differences in the VAS scores of dry mouth in patients among the four groups were analyzed using a two-way repeated measurement ANOVA with Greenhouse–Geisser correction, and the Bonferroni correction test was used for further statistical comparisons between groups.

* *p* < 0.008 vs. NS group

^#^ *p* < 0.005 vs. 1 h before surgery.

The postoperative nausea VAS scores in four groups at different time points are shown in Table 7. There was no difference in postoperative nausea VAS scores among the four groups at 2, 8, 24, and 48 h after surgery (*p* = 0.162, 0.381, 0.266, and 0.983, respectively). With the prolongation of time after surgery, the VAS score for postoperative nausea in the four groups decreased gradually (all *p* < 0.001).

**TABLE 7:** Postoperative nausea VAS score in the four groups.

Time points	A group (*n* = 144)	G group (*n* = 144)	P Group (*n* = 144)	NS group (*n* = 144)	*χ* ^*2*^ values	*p*-values
2 h after surgery	2 (2, 2)	2 (1, 2)	2 (1, 2)	2 (1, 2)	5.140	0.162
8 h after surgery	2 (2, 3)	2 (1, 3)	2 (2, 3)	2 (1, 3)	3.067	0.381
24 h after surgery	0 (0, 1)	0 (0, 1)	0 (0, 1)	0 (0, 1)	3.958	0.266
48 h after surgery	0 (0, 0)	0 (0, 0)	0 (0, 0)	0 (0, 0)	0.168	0.983
*χ* ^*2*^ values	350.346	319.080	348.132	286.329	-	-
*P*-values	<0.001	<0.001	<0.001	<0.001	-	-

Values are presented as the median [interquartile range (IQR)]. A group, atropine group; G group, glycopyrrolate group; P group, penehyclidine group; NS group, normal saline group. Differences in the postoperative nausea VAS scores in patients among four groups were analyzed using the Kruskal–Wallis test.

The severity of postoperative vomiting in the four groups is shown in Table 8. There was no difference in the severity of vomiting at 2, 8, 24, and 48 h after surgery among the four groups (*p* = 0.738, 0.565, 0.986, and 0.797, respectively).

**TABLE 8:** Severity of postoperative vomiting of patients in the four groups.

Time point	Group	n	0	1	2	3	*χ* ^2^ values	*p-*values
2 h after surgery	A group	144	131	13	0	0	1.325	0.738
	G group	144	135	9	0	0		
	P group	144	130	14	0	0		
	NS group	144	132	12	0	0		
8 h after surgery	A group	144	92	41	11	0	4.839	0.565
	G group	144	108	30	6	0		
	P group	144	102	34	8	0		
	NS group	144	104	32	8	0		
24 h after surgery	A group	144	129	12	3	0	1.148	0.986
	G group	144	127	13	4	0		
	P group	144	127	12	5	0		
	NS group	144	125	15	4	0		
48 h after surgery	A group	144	137	7	0	0	1.187	0.797
	G group	144	138	6	0	0		
	P group	144	134	10	0	0		
	NS group	144	136	8	0	0		

Values are presented as the number of patients. A group, atropine group; G group, glycopyrrolate group; P group, penehyclidine group; NS group, normal saline group. Differences in the severity of postoperative vomiting among the four groups were analyzed using *Chi-*square tests or Fisher’s exact tests.

## Discussion

The results of this study indicated that there was a significant increase in patients who developed POD 24 h after surgery in the atropine and penehyclidine groups, and there was no difference between the G and NS groups in the incidence of POD at 24 h after surgery. Oral saliva secretion in patients significantly decreased within 8 h after premedication with atropine, glycopyrrolate, and penehyclidine. At 24 h after surgery, the QoR-15 scale score of patients in the glycopyrrolate group was significantly higher than that in the atropine group. There was no difference in the incidence of postoperative nausea and vomiting after premedication with atropine, glycopyrrolate, and penehyclidine.

Postoperative delirium is an acute neuropsychiatric syndrome that occurs after surgery, which delays postoperative recovery and increases mortality and health care costs (Li et al., 2022). A questionnaire survey of 1,780 older patients aged more than 70 years showed a significant correlation between the use of anticholinergic drugs and cognitive impairment in community-dwelling older people (Lechevallier-Michel et al., 2005). Ferré et al. (2022) reported that the administration of atropine caused POD in approximately 54% of elderly patients undergoing hip replacement surgery. In the present study, after intramuscular injection of atropine 30 min before surgery, 20.1% of older patients developed POD, indicating that atropine as a premedication can lead to POD in elderly patients after surgery. However, the incidence of POD in the older patients in the above study was significantly higher than that in this study. This is mainly because the participants in this study underwent submucosal dissection, which causes less trauma to the patients. However, patients in the above study underwent hip replacement, which causes greater trauma to the patients, resulting in a higher incidence of POD in older patients (Price et al., 2008). Clinical studies have found that the use of penehyclidine as a premedication can reduce the incidence of postoperative pulmonary complications by reducing pro-inflammatory cytokines but increase the incidence of postoperative cognitive impairment and postoperative delirium in older patients with cancer undergoing thoracoscopic surgery under general anesthesia (Hongyu et al., 2019). We administered penehyclidine as a premedication for older patients undergoing submucosal dissection, and 23.6% of older patients had POD. It is suggested that penehyclidine as a premedication can lead to postoperative delirium in older patients (Yao et al., 2020). A meta-analysis of 138 clinical trials found that the administration of glycopyrrolate did not lead to postoperative delirium in patients (Hasselmo, 2006). In our study, the incidences of POD in the glycopyrrolate and normal saline groups were similar (10.45% vs. 9%), indicating that glycopyrrolate as a premedication for elderly patients does not lead to postoperative delirium, which is consistent with the results of the above meta-analysis.

Acetylcholine is one of the central neurotransmitters involved in human cognitive activities, and blocking its effect can lead to cognitive dysfunction in patients (Naseri et al., 2023). Both atropine and penehyclidine can penetrate the blood–brain barrier and act on the central M receptor, resulting in cognitive dysfunction in patients (Hasselmo, 2006). Glycopyrrolate cannot penetrate the blood–brain barrier and does not affect central M receptors; therefore, it does not interfere with patients’ cognitive function (Naseri et al., 2023). Inflammation plays a crucial role in postoperative cognitive dysfunction in elderly patients (Rump and Adamzik, 2022). Clinical studies have shown that penehyclidine can inhibit the release of inflammatory mediators and reduce the inflammatory response (Tan et al., 2022; Weng et al., 2023; Wang et al., 2018). However, POD still occurred in older patients in the penehyclidine group in this study, suggesting that the anti-inflammatory effect of penehyclidine could not improve the cognitive dysfunction caused by its action on the central M receptor. Glycopyrrolate can not only reduce the level of plasma inflammatory mediators and improve tissue inflammation in patients with acute respiratory distress syndrome (Liu et al., 2022) but also lower the expression levels of IL-4, IL-5, IL-6, and IL-13 in bronchial tissue *in vitro* (Rogliani et al., 2021). At present, few studies have investigated the effect of glycopyrrolate on the inflammatory response (Calzetta et al., 2021), especially its effect on inflammation in the central nervous system. The relationship between the effect of glycopyrrolate on inflammation and POD remains unclear.

Anticholinergic drugs inhibit salivary gland secretion by blocking M receptors, leading to a decrease in saliva secretion and causing postoperative dry mouth in patients (Hilmer and Gnjidic, 2022). Two groups of patients who underwent oral surgery under local anesthesia were intramuscularly injected with 0.6 mg atropine and 0.2 mg glycopyrrolate at 30 min before surgery, respectively. The oral saliva secretion of the two groups was significantly inhibited at the beginning of the surgery (Rachana and Sequeira, 2020). At 30 min before anesthesia, two groups of patients undergoing gynecological laparoscopic surgery were intravenously injected with atropine at 0.01 mg/kg and penehyclidine at 0.01 mg/kg, respectively. Postoperative oropharyngeal secretions in the two groups were significantly reduced, accompanied by an increased incidence of dry mouth (Ye et al., 2020). In this study, the salivary secretion of patients in the atropine, glycopyrrolate, and penehyclidine groups was significantly decreased from before anesthesia to 8 h after surgery, and the incidence of dry mouth in the three groups was significantly increased from 2 h to 8 h after surgery, which was consistent with the above clinical study results. Although patients in the NS group did not receive premedication, oral salivary secretion was slightly reduced at T_1_–T_5_. This may be due to preoperative fasting from solid food and drinking. When atropine and penehyclidine were administrated as premedication, oral secretions in the penehyclidine group were found to be significantly reduced compared with those in the atropine group (Pihlajamäki et al., 1986). During fiberoptic bronchoscopy, the administration of atropine (0.01 mg/kg) was found to be more effective in reducing oral and airway secretions than glycopyrrolate (0.005 mg/kg) when administered 30 min before surgery (Malik et al., 2009). In the present study, the oral secretions of patients in the penehyclidine and glycopyrrolate groups were also significantly lower than those in the atropine group from 30 min after administration to 8 h after surgery. This shows that glycopyrrolate and penehyclidine as premedication are more effective in reducing oral secretions than atropine. In addition, the duration of decreased saliva secretion and dry mouth in the penehyclidine group was found to be significantly longer than that in the atropine and glycopyrrolate groups, which also led to a significant decrease in the QoR-15 score in the penehyclidine group at 24 h after surgery. This may be mainly due to the significantly shorter elimination half-lives of atropine and glycopyrrolate compared to penehyclidine (Lu et al., 2022; Sechaud et al., 2016; Müller et al., 2012), resulting in a significant prolongation of saliva secretion inhibition and dry mouth in patients in the penehyclidine group.

Clinical studies have found that compared with the saline control group, penehyclidine did not reduce the incidence or severity of PONV in patients undergoing laparoscopic bariatric surgery (Ding et al., 2023). Compared with the normal saline control group, neostigmine (0.05 mg/kg) and glycopyrrolate (0.01 mg/kg) can increase the incidence of postoperative nausea and vomiting in patients undergoing laparoscopic gynaecological surgery (Løvstad et al., 2001). However, Tait et al. (2007) reported that children who were administered 0.01 mg/kg glycopyrrolate showed a significant reduction in postoperative nausea and vomiting. This indicated that there is an inconsistency in the effect of glycopyrrolate on postoperative nausea and vomiting. In our study, the incidence and severity of postoperative nausea and vomiting were comparable with the use of atropine, glycopyrrolate, and penehyclidine as premedications.

There are several limitations in this study. First, postoperative neuroinflammation is a critical pathogenic mechanism for postoperative cognitive dysfunction (Alam et al., 2018). Although it has been reported that penehyclidine and glycopyrrolate have certain anti-inflammatory effects, the postoperative levels of inflammatory mediators and neuroinflammation were not assessed in patients in this study. Therefore, the effects of penehyclidine and glycopyrrolate on postoperative neuroinflammation remain unclear and need to be investigated in future studies. Second, postoperative delirium often occurs in 10%–60% of older patients undergoing major surgery and is associated with prolonged hospital stay and increased hospitalization costs (Deiner et al., 2017). In the present study, we only observed the effects of three anticholinergic drugs on POD in patients undergoing gastric or esophageal submucosal dissection. The endoscopic submucosal dissection (ESD) procedure was relatively less traumatic, and patients recovered quickly after surgery. The effect of different anticholinergic drugs as premedication on POD in elderly patients undergoing major surgery remains unclear. Third, a clinical study found that preoperative intramuscular injection of 0.005 mg/kg glycopyrrolate can lead to an increase in postoperative body temperature in pediatric patients (Kim et al., 2016). In our study, no postoperative increase in body temperature was observed in older patients. This may be because children are more likely to have elevated body temperatures after anticholinergic injections than older patients (Kim et al., 2016).

In conclusion, glycopyrrolate is more suitable as a premedication for older patients without aggravating postoperative delirium or prolonging dry mouth.

## Data Availability

The original contributions presented in the study are included in the article/supplementary material, further inquiries can be directed to the corresponding author.
